# [Mo_3_S_13_]^2−^ as a Model System for Hydrogen Evolution Catalysis by MoS_x_: Probing Protonation Sites in the Gas Phase by Infrared Multiple Photon Dissociation Spectroscopy

**DOI:** 10.1002/anie.202014449

**Published:** 2021-01-26

**Authors:** Aristeidis Baloglou, Manuel Plattner, Milan Ončák, Marie‐Luise Grutza, Philipp Kurz, Martin K. Beyer

**Affiliations:** ^1^ Institut für Ionenphysik und Angewandte Physik Universität Innsbruck Technikerstraße 25 6020 Innsbruck Austria; ^2^ Institut für Anorganische und Analytische Chemie und Freiburger Materialforschungszentrum (FMF) Albert-Ludwigs-Universität Freiburg Albertstraße 21 79104 Freiburg Germany

**Keywords:** hydrogen evolution reaction, IRMPD spectroscopy, mass spectrometry, molybdenum sulfide clusters

## Abstract

Materials based on molybdenum sulfide are known as efficient hydrogen evolution reaction (HER) catalysts. As the binding site for H atoms on molybdenum sulfides for the catalytic process is under debate, [HMo_3_S_13_]^−^ is an interesting molecular model system to address this question. Herein, we probe the [HMo_3_S_13_]^−^ cluster in the gas phase by coupling Fourier‐transform ion‐cyclotron‐resonance mass spectrometry (FT‐ICR MS) with infrared multiple photon dissociation (IRMPD) spectroscopy. Our investigations show one distinct S−H stretching vibration at 2450 cm^−1^. Thermochemical arguments based on DFT calculations strongly suggest a terminal disulfide unit as the H adsorption site.

## Graphical abstract

The cluster compound [Mo_3_S_13_]^2−^ is an interesting model system for hydrogen evolution catalysis. Herein, we probe the protonation site of the cluster by coupling mass spectrometry with infrared action spectroscopy. One distinct band at 2450 cm^−1^ together with DFT calculations and IRMPD kinetics reveals the nature of this S−H stretching vibration.

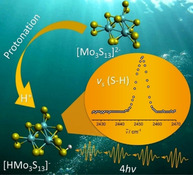

By utilizing electrochemical water‐splitting, renewable energy sources, such as solar or wind power, can be stored in the form of molecular hydrogen (H_2_).^[1]^ Hydrogen can be used directly as a fuel,^[2]^ serves as a valuable feedstock for chemical syntheses^[3]^ and can also replace carbon as reducing agent in steel production.^[4]^ Commercial electrolyzers for water splitting currently often rely on platinum as a catalyst for the hydrogen evolution reaction (HER), especially when the proton‐exchange membrane (PEM) technology is used at low pH.^[5]^ Due to the limited availability and high cost of platinum, the search for precious metal‐free and acid‐stable HER catalysts is a very active research field.^[6]^ Nanostructured molybdenum sulfides represent an attractive class of compounds to make hydrogen production economically viable, since they show promising activities and stabilities especially for the pH range between pH 0 and pH 7.[^7^, ^8^] Among the most active forms of the material are amorphous, non‐stoichiometric molybdenum sulfides (MoS_*x*_).[^9^, ^10^]

Amorphous molybdenum sulfides are often described as polymeric chains, which contain fragments derived from the triangular binding motif present in the thiomolybdate cluster [Mo_3_S_13_]^2−^.[^11^, ^12^] The exact reaction mechanism of proton reduction by MoS_*x*_ is still under debate. Tran et al. propose undercoordinated Mo moieties as HER active sites, which form under catalytic conditions. They undergo protonation under HER conditions resulting in Mo hydride (Mo‐H) intermediates.^[12]^ In a different study, Lassalle‐Kaiser et al. suggest the involvement of terminal disulfide units in catalytic proton reduction.^[13]^ The latter is supported by operando Raman spectroscopy, pinpointing the sulfur atoms as catalytically active sites.^[14]^ Furthermore, by studying molecular mimics of MoS_2_, Wu and co‐workers found evidence of sulfide ligands acting as redox center.^[15]^ Thiomolybdate nanoclusters, like [Mo_3_S_13_]^2−^ and [Mo_2_S_12_]^2−^, were found to exhibit excellent activities as HER electrocatalysts when deposited on electrodes.^[16]^ More recently, both clusters were also found to catalyze visible light‐driven HER in homogeneous systems.[^17^, ^18^] In the case of [Mo_3_S_13_]^2−^, an exchange of the terminal disulfides was found to modulate the catalyst's redox‐activity.^[18]^ However, in investigations of electrodes coated with [Mo_3_S_13_]^2−^ clusters, Yeo et al. suggest that, under catalytic conditions, the bridging S_2_^2−^ might be of higher reactivity than the terminal ones.^[19]^

In order to facilitate mechanistic investigations on this very interesting system for HER catalysis, we utilize a bottom‐up approach and study [Mo_3_S_13_]^2−^ as a precisely defined, molecular model for MoS_*x*_ in the gas phase. Previous gas phase studies have shed light on intrinsic properties of molybdenum sulfide and oxide clusters,[^20^, ^21^] in particular their reactivity with hydrogen and water.^[22]^ Our goal is to identify intrinsic properties of the clusters associated with HER activity. The trinuclear [Mo_3_S_13_]^2−^ cluster features three different sulfur moieties: the apical sulfur atom (μ_3_‐S^2−^) at the center of the molecule, three bridging disulfide ligands (*η* ^2^‐*μ*‐S_2_^2−^) connecting the three Mo‐atoms, and three side‐on coordinated terminal disulfide ligands (*η* ^2^‐S_2_^2−^) (see Figure S1).^[9]^ In a previous study, we investigated the geometries and structural flexibility of mass selected, isotopically enriched [^92^Mo_3_S_13_]^2−^ as well as the singly and triply protonated ions, [H^92^Mo_3_S_13_]^−^ and [H_3_^92^Mo_3_S_13_]^+^, respectively, in the gas phase using collision induced dissociation (CID).^[21]^ The protonated thiomolybdate clusters are of particular interest, as they serve as models for the hydrogen (H^+^ + e^−^) adsorption intermediate during HER catalysis (Volmer reaction).^[23]^

We now probe the protonation site of the clusters experimentally with infrared multiple photon dissociation (IRMPD) spectroscopy.[^24^, ^25^] The mass‐selected, singly‐protonated cluster [HMo_3_S_13_]^−^ was irradiated with tunable laser light to acquire its IRMPD spectrum. During an IRMPD event, a vibrational mode is resonantly excited by photons and the complex is successively heated by intramolecular vibrational redistribution (IVR), inducing dissociation.^[24]^ The fragment ions are then detected by mass spectrometry. Absolute IR absorption cross sections are derived from the measured photodissociation yield as described previously.[^26^, ^27^] Briefly, sequential photon absorption can be described by a series of first‐order reactions, assuming that the absorption cross section stays constant and radiative cooling is negligible.

An IRMPD spectrum of [HMo_3_S_13_]^−^ (Figure 1) recorded in the range of 2245–2660 cm^−1^ with a step‐size of 3 cm^−1^ revealed a single, relatively sharp band at 2450 cm^−1^. The measured frequency is typical of S‐H stretching vibrations.^[28]^ To rule out the presence of Mo‐H containing isomers, we also performed IRMPD spectroscopy in the range of 1600–2000 cm^−1^. Within this spectral region, no cluster fragmentation was detected. By utilizing matrix IR spectra, Andrews showed that MoH_*x*_ (*x=*2, 4, 6) species exhibited absorption bands in the range of 1700–1850 cm^−1^.^[29]^ Hence our spectral data are consistent with the absence of Mo‐H containing isomers.[^13^, ^14^, ^19^]

**Figure 1:**
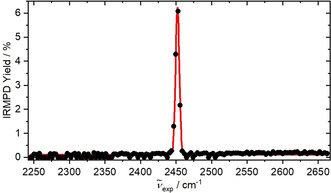
Action spectrum showing the IRMPD yield in the range of the different S‐H stretching vibrations. Only one sharp absorption band was observed, indicating the presence of only one isomer in the experiment.

In order to determine which protonation sites are present, we performed quantum chemical calculations on the protonated cluster [HMo_3_S_13_]^−^ using density functional theory (DFT). The different sulfur moieties provide four distinct protonation sites as shown in Figure 2: two terminal isomers (**T1**, **T2**), with the proton bound to either sulfur atom, respectively, apical (**A**) and bridging (**B**) isomers as well as an isomer in which the Mo_3_ ring opens (**O**). Clusters with protonated terminal groups are the most stable, with apical and bridging isomers lying more than 1 eV higher in energy. The difference in energy between isomers with the proton located on different sulfur atoms of the terminal S_2_ group is about 0.1 eV and, within the error limits of the DFT approach, both isomers are thus expected to be present in the experimental mixture.

**Figure 2:**
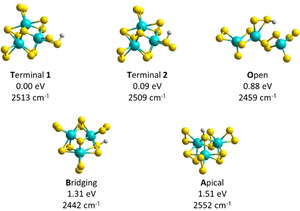
Relevant structures of [HMo_3_S_13_]^−^ with the proton located at different S moieties, along with relative energies and harmonic frequency of the S‐H mode (scaled by 0.957). Calculated at the ωB97XD/aug‐cc‐pVTZ(‐PP) level of theory.

The relative position of the S‐H vibration present in all isomers is consistent among various DFT functionals. Within the precision of the DFT approach, however, the calculations do not allow for pinpointing the isomer based on the position of the IR vibration (see also the SI for benchmark against CCSD and MP2 methods). Relative isomer stability, however, suggests that only the terminal isomers are present.

Let us now consider the thermodynamics of the observed dissociation. In our previous study, we calculated the dissociation energy of an S_2_ unit from the protonated cluster [HMo_3_S_13_]^−^ as 1.47 eV, that is, about 11,800 cm^−1^.^[21]^ With the photon energy of 2452 cm^−1^ in the measured spectral region, about five photons are needed to provide this dissociation energy at 0 K. Since the experiments are performed at room temperature, the clusters already have considerable internal energy, calculated at 0.62 eV for **T1**; therefore, four photons should be sufficient for dissociation, if radiative cooling is inefficient.

To test this hypothesis, we conducted IRMPD kinetic studies, where the laser wavelength is fixed to the maximum of the absorption band and the irradiation time is gradually increased from 0 s to 30 s, as shown in Figure 3 a. As expected for a multiple photon process, the data shows a relatively high induction time interval of 1.5 s.

**Figure 3:**
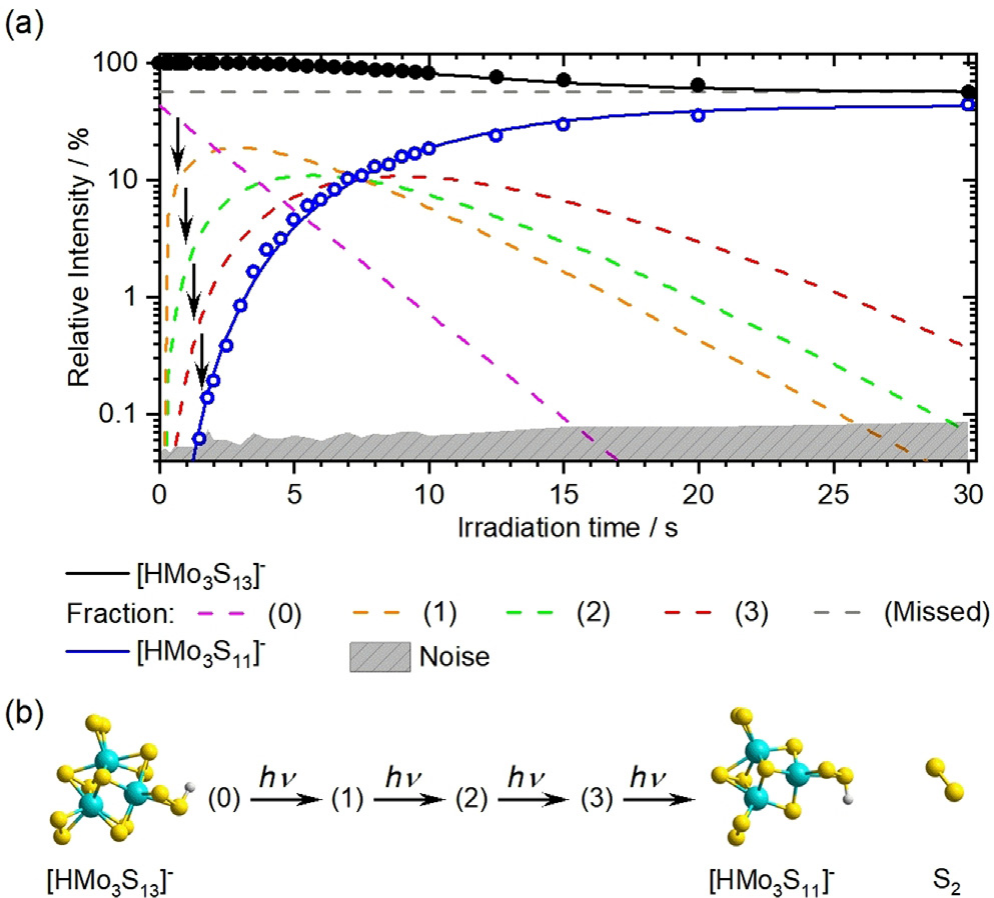
(a) IRMPD kinetics for [HMo_3_S_13_]^−^ → [HMo_3_S_11_]^−^ + S_2_ dissociation. The experimental data is shown with black (precursor) and blue (fragment) circles and the kinetic fit with lines. Dashed lines represent precursor fractions. The sum of the fractions equals the measured precursor intensity. (b) Depiction of the implemented four‐photon kinetic model.

In order to quantitatively model the data, the signal of the precursor ion was described by four fractions, the original ions and ions having absorbed 1, 2 or 3 IR photons. The analysis was performed assuming first order kinetics for the transition from one fraction to the next upon absorption of an IR photon. The cascade (0)‐(3) corresponds to the sequential heating of the precursor, with the fourth photon leading to the observed fragmentation (see Figure 3 b).

After 30 s of irradiation, about 55 % of the precursor ions remain intact, thus an additional “*missed”* fraction can be interpreted as a partly irradiated ion cloud or as the presence of another isomer that does not resonantly absorb in this region. The latter could be rationalized via an isomerization, in which an isomer is formed below the dissociation threshold, that does not resonantly absorb at 2452 cm^−1^. This is a well‐established effect in IRMPD of hydrogen bonded systems.^[30]^

We also tested this hypothesis with the ring‐opened isomer **O**, lying 0.88 eV higher than the most stable **T1** isomer (Figure 2).^[21]^ The S−H bond in the **O** isomer is calculated to absorb at about 2459 cm^−1^, about 50 cm^−1^ redshifted from the absorption of the **T1**, **T2** structures. To test whether the **O** isomer is significantly populated near the dissociation threshold, we calculated the relative contribution of all isomers from Figure 2 to the total density of states as a function of energy,^[31]^ displayed in Figure S2. This calculation clearly shows that essentially only the **T1**, **T2** isomers contribute to the population near the dissociation threshold. It is therefore more plausible that the laser beam has insufficient overlap with the ion cloud, that is, only 45 % of the ions are actually irradiated.

The quality of the kinetic fit indicates that a four‐photon process is a realistic description of the experiment, which does not rule out that some ions fragment after absorption of 3 or 5 photons. The rate of photon absorption together with the photon flux calculated from laser power and beam diameter yields IR absorption cross sections on the order of 5×10^−20^ cm^2^, matching the calculated values within the error limits of the experiment.[^26^, ^27^]

In order to compare theory to experiment, we measured the observed absorption band again in more detail with a step size of 0.6 cm^−1^, revealing the shape of the band at 2452 cm^−1^ with a full width at half maximum (FWHM) of 12 cm^−1^. Figure 4 a shows a comparison between the theoretical absorption bands employing the ωB97XD functional and the measured spectrum in the S‐H stretching region. The theoretical bands were broadened with Gaussian functions employing aFWHM of 10 cm^−1^. The experimental cross sections are shown for a four‐photon process. Based on the calculated thermochemistry, we expect to observe isomers with a protonated terminal disulfide unit. Since **T1** and **T2** lie only about 0.1 eV apart, it is reasonable to assume the existence of both isomers in the experiment, supported by the density of states calculations. Therefore, as shown in Figure 4 b, the experimental data were modeled by two Gaussian functions representing the two isomers (see Table S1). Indeed, the theoretical and fitted Gaussians are in good agreement, consistent with the existence of two absorption bands contributing to the total measured cross section. The relative intensity, however, deviates from the calculated density of states, albeit within the error limits of the calculated thermochemical values.

**Figure 4:**
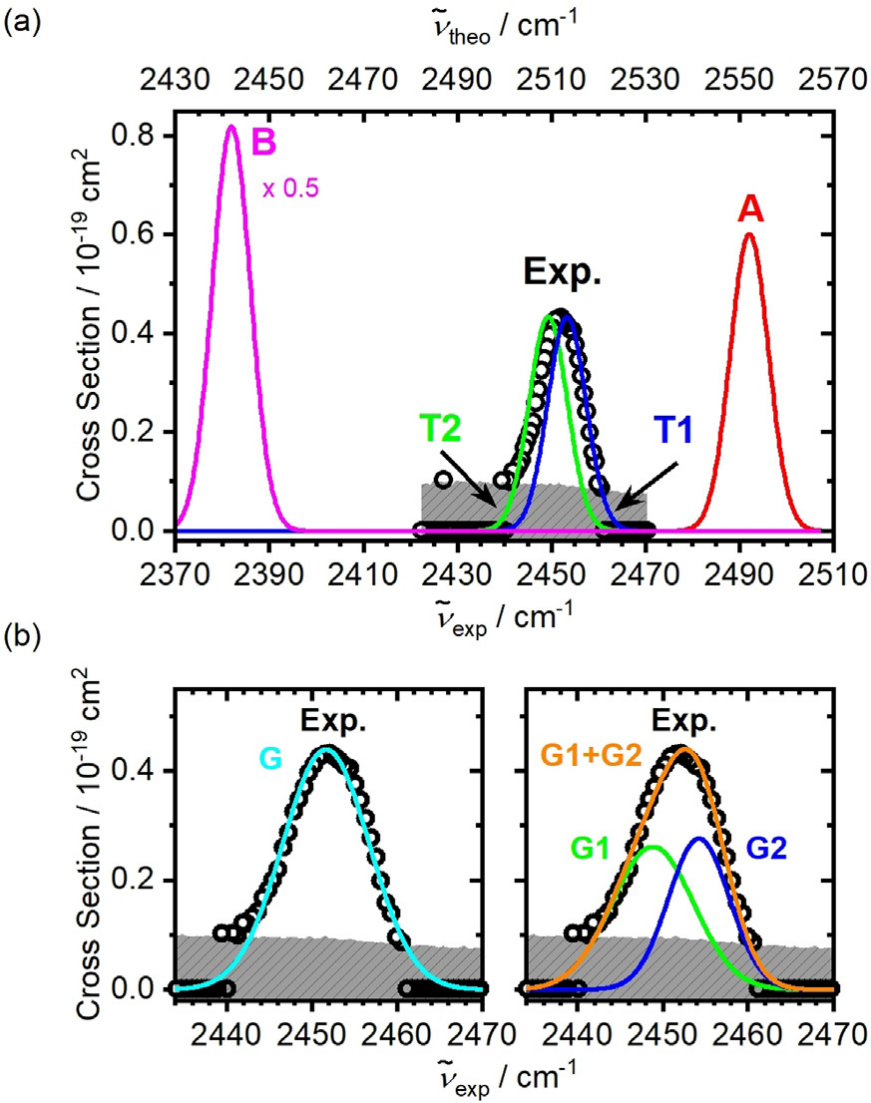
(a) Calculated IR spectra for the relevant structures at the ωB97XD/aug‐cc‐pVTZ(‐PP) level of theory (the full width at half maximum was chosen as 10 cm^−1^) along with the experimental IRMPD spectrum assuming a 4‐photon process. (b) Magnified spectrum of the measured absorption band assuming a 4‐photon process and fitted using one (left) and two (right) Gaussian functions.

Our experimental results thus clearly show that protonation of intact [Mo_3_S_13_]^2−^ clusters occurs at a sulfur moiety. Thermochemically, protonation at terminal disulfide groups is favored over bridging and apical sites by more than 1 eV. Obviously, these results from gas‐phase clusters are not directly transferable to electrochemical reactions in bulk aqueous solution, since solvation can have a substantial effect on the energetics of ionic reaction pathways. Nevertheless, our results reveal the intrinsically preferred proton site in a molybdenum sulfide model catalyst. It thus supports a sulfur‐centered mechanism for HER catalysis by MoS_*x*_ where proton‐binding can be considered as key reaction step.[^13^, ^14^, ^18^, ^19^] Further investigations have to show whether the same conclusion can be drawn for clusters where one or more of the terminal S_2_^2−^ groups have been removed as described (and considered to be beneficial for HER catalysis) by Dave et al.^[18]^

## Conflict of interest

The authors declare no conflict of interest.

## Supporting information

As a service to our authors and readers, this journal provides supporting information supplied by the authors. Such materials are peer reviewed and may be re‐organized for online delivery, but are not copy‐edited or typeset. Technical support issues arising from supporting information (other than missing files) should be addressed to the authors.

Supplementary
