# Supplementary material for: [Mo3S13]2− as a Model System for Hydrogen Evolution Catalysis by MoSx: Probing Protonation Sites in the Gas Phase by Infrared Multiple Photon Dissociation Spectroscopy
Source: Angew Chem Int Ed Engl. 2021 Jan 26;60(10):5074–7. doi: 10.1002/anie.202014449 (PMC7986116; doi:10.1002/anie.202014449)
Supplement: Supplementary file 1 — Supplementary [file ANIE-60-5074-s001.pdf]

Angewandte Chemie
Eine Zeitschrift der Gesellschaft Deutscher Chemiker

Supporting Information

$[Mo_3S_{13}]^{2-}$ as a Model System for Hydrogen Evolution Catalysis by MoS_x: Probing Protonation Sites in the Gas Phase by Infrared Multiple Photon Dissociation Spectroscopy

Aristeidis Baloglou, Manuel Plattner, Milan Ončák, Marie-Luise Grutza, Philipp Kurz, and Martin K. Beyer**

anie_202014449_sm_miscellaneous_information.pdf

Supporting Information

©Wiley-VCH 2020
69451 Weinheim, Germany

Abstract: Molybdenum sulfide based materials are known as efficient hydrogen evolution reaction (HER) catalysts. As the binding site for H atoms on molybdenum sulfide for the catalytic process is under debate, $[HMo_3S_{13}]^-$ is an interesting molecular model system. Herein, we probe the $[HMo_3S_{13}]^-$ cluster in the gas phase by coupling Fourier transform ion cyclotron resonance mass spectrometry (FT-ICR MS) with infrared multiple photon dissociation (IRMPD) spectroscopy. Our investigations show one distinct S-H stretching vibration at 2450 cm^{-1}. Thermochemical arguments based on DFT calculations strongly suggest a terminal disulfide unit as the H adsorption site.

DOI: 10.1002/anie.202014449 and 10.1002/ange.202014449

Table of Contents

Experimental Section 2
[Mo_3S_{13}]$^{2–}$ Geometry 2
Additional Experimental Data 3
Additional Computational Data 3
References 7
Author Contributions 7

Experimental Section

The experimental setup has been described in detail previously.[1,2] Photodissociation spectroscopy was performed in ultrahigh vacuum (≈ 10^{-9} mbar) inside the ICR cell of a commercial Bruker Apex Qe FT-ICR MS, equipped with a combined electrospray ionization (ESI) and matrix assisted laser desorption ionization (MALDI) Apollo II Dual ESI/MALDI ion source along with a 9.4 T superconducting magnet. Tuneable IR laser light from optical parametric oscillators (OPO) EKSPLA NT277 and NT273, operated at a 1 kHz repetition rate, is guided through a CaF_2 window at the rear end of the vacuum chamber into the ICR cell.[1,2] Electrospray ionization of $(NH_4)_2{}^{92}Mo_3S_{13}$ dissolved in water-methanol (1:1) yields mainly [Mo_3S_{13}]$^{2-}$ and [HMo_3S_{13}]$^{-}$. Subsequently the protonated clusters [HMo_3S_{13}]$^{-}$ were preselected by a quadrupole mass filter and guided into the ICR cell, where they were trapped for irradiation. The IR photodissociation ratio was obtained by recording a mass spectrum at each set wavelength, yielding an IR action spectrum. The wavelength of the OPO was calibrated with an accuracy of <2 nm by a laser spectrum analyser Ångstrom LSA IRIII L (HighFinesse). $(NH_4)_2$[$^{92}Mo_3S_{13}$] was prepared from isotopically enriched $^{92}MoO_3$ (STB Isotope Germany), following a procedure of Müller et al.,[3] as described previously.[4] Quantum chemical calculations were performed using the ωB97XD functional, with the vibrational scaling factor of 0.957 (see the SI for benchmark calculations). The Gaussian software was used for calculations.[5]

[Mo_3S_{13}]$^{2–}$ Geometry

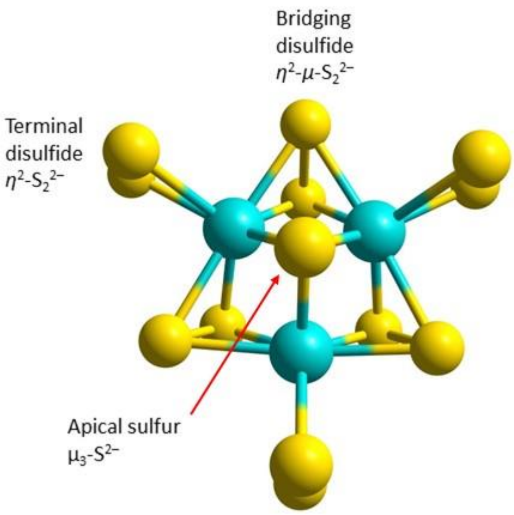

Figure S1. Different sulfur moieties of the [Mo_3S_{13}]$^{2-}$ cluster.

Additional Experimental Data

Table S1. Gauss functions used to model the experimental data in Figure 3b. Fitting model: $G(x) = \frac{A}{w\sqrt{\pi/2}} e^{-2\left(\frac{x-x_c}{w}\right)^2}$

	x_c / cm^{-1}	w / cm^{-1}	A / 10^{-19} cm	R^2
G	2451.7 ± 0.1	10.2 ± 0.2	5.6 ± 0.1	0.974
G1	2449 ± 5	10 ± 3	3.1 ± 3.4	0.984
G2	2454 ± 1	7 ± 2	2.5 ± 3.4	

Additional Computational Data

Table S2 shows calculations of S–H vibrations for several molecules containing S–H bonds. Based on this calculations, we can conclude that scaling factors of 0.968 and 0.957 for B3LYP and ωB97XD from NIST[6] lie within 1% of the values calculated for the tested molecules and are therefore used also in our calculations. For the M06, we pick the factor of 0.967.

Table S2. Unscaled S–H vibrational frequencies in several molecules along with experiment (in cm^{-1}). The aug-cc-pVTZ basis set was used for all calculations.

	CCSD	B3LYP	M06	ωB97XD	exp.
H_2S	2727.9; 2742.8	2679.0; 2692.1	2695.7; 2705.9	2743.7; 2756.2	2614.6; 2626 [7]
H_2S_2	2696.2; 2697.8	2627.5; 2629.6	2639.2; 2642.2	2689.4; 2691.2	2553.8; 2556.6 [8]
CH_3SH	2742.8	2674.3	2693.1	2735.5	2597 [9]

Table S3. Relative energies ΔE (in eV) and vibrational frequencies v (in cm^{-1}) calculated for various $[HMO_3S_{13}]^-$ isomers using the aug-cc-pVTZ(-PP) basis set. Scaling factors of 0.968 (B3LYP), 0.967 (M06) and 0.957 (ωB97XD) were used.

	ΔE			v		
Isomer	B3LYP	M06	ωB97XD	B3LYP	M06	ωB97XD
**A**	1.26	1.31	1.31	2477	2506	2552
**T1**	0.00	0.00	0.00	2483	2478	2513
**T2**	0.10	0.09	0.09	2479	2489	2509
**B**	1.27	1.39	1.51	2409	2411	2442

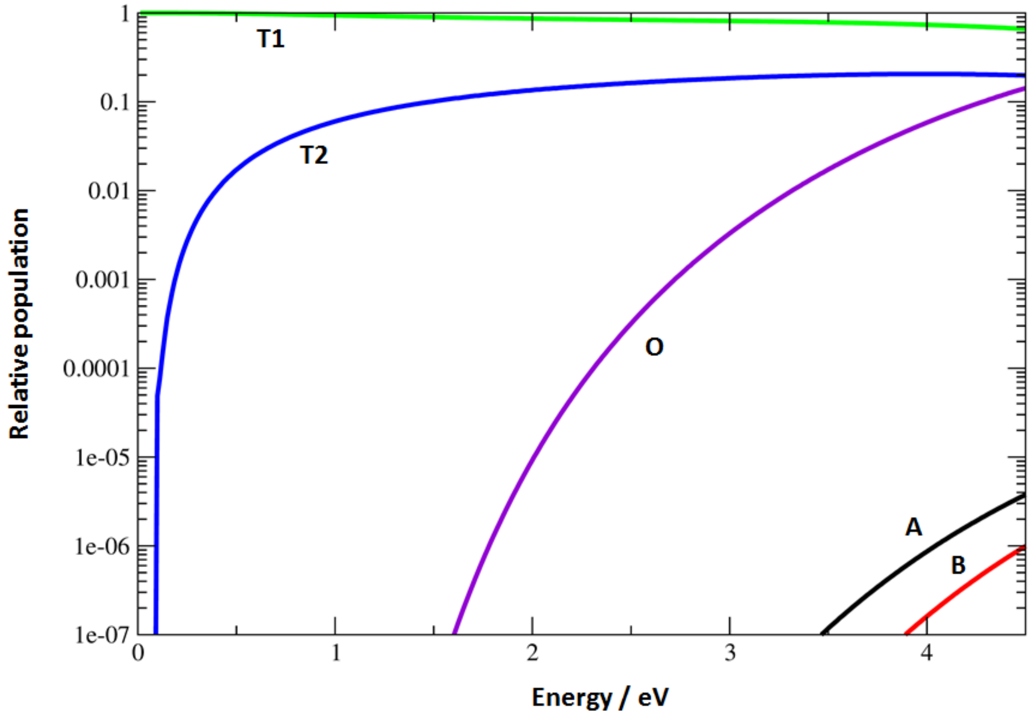

Figure S2. Relative population of various $[HMo_3S_{13}]^-$ isomers calculated through the density of states at the B3LYP/aug-cc-pVTZ(-PP) level of theory.

Cartesian coordinates (in Å) and electronic energies (in a.u.) of various $HMo_3S_{13}^-$ structures optimized using various DFT functionals; see methods for more computational details

Isomer A, wB97XD
E= -5381.897509
S 0.000239 -0.000415 1.776810
Mo -1.498590 -0.530315 0.031039
S -1.440442 1.229234 -1.625026
S -2.199108 1.878991 0.138971
Mo 0.289787 1.562598 0.030637
S 2.726452 0.965191 0.136853
S 1.784089 0.631809 -1.626596
Mo 1.209018 -1.032334 0.030321
S -0.345025 -1.859484 -1.625758
S -0.527213 -2.844038 0.136658
S 0.652128 3.510800 1.391252
S 0.718950 3.878458 -0.640770
S 3.000020 -2.562659 -0.640232
S 2.715182 -2.318846 1.391729
S -3.719000 -1.316373 -0.641235
S -3.367037 -1.192476 1.390858
H 0.003216 -0.000961 3.119923

Isomer T2, wB97XD
E= -5381.942227
S 0.022032 -0.002060 1.807039
Mo -1.529411 -0.004816 -0.004760
S -0.963314 1.672129 -1.640744
S -1.440509 2.477777 0.165665
Mo 0.799332 1.350548 0.011270
S 2.885296 0.009220 0.086197
S 1.902615 0.006634 -1.691818
Mo 0.808950 -1.345660 0.010942
S -0.948032 -1.674478 -1.644105
S -1.425198 -2.489506 0.160282
S 1.768567 2.981633 1.469582
S 2.008564 3.424358 -0.538861
S 2.032135 -3.412205 -0.537404
S 1.787558 -2.969824 1.470690
S -3.976551 -0.024272 -0.640639
S -3.628344 0.076936 1.380163
H -3.709697 -1.224538 1.730308

Isomer T1, wB97XD
E= -5381.945514
S -0.015327 -0.005581 1.799838
Mo 1.516065 -0.249743 0.001894
S 0.662319 -1.788840 -1.665087
S 1.016218 -2.698581 0.125139
Mo -1.006593 -1.208731 0.006213
S -2.844432 0.453379 0.112355
S -1.899505 0.301988 -1.678890
Mo -0.578755 1.451464 0.009572
S 1.186283 1.478425 -1.665070
S 1.821417 2.231330 0.123141
S -2.215245 -2.668318 1.469364
S -2.548537 -3.046655 -0.538547
S -1.466978 3.681871 -0.530626
S -1.264005 3.212545 1.475858
S 4.019650 -0.631938 -0.505112
S 3.457588 -0.542330 1.476205
H 4.358726 0.657651 -0.719579

Isomer B, wB97XD
E= -5381.890017
S -0.097476 0.026825 -1.832585
Mo 1.334098 -0.728703 -0.046263

S 2.183395 0.582482 1.968813
S 2.366707 1.568415 0.152389
Mo -0.129773 1.598307 -0.096350
S -2.585381 1.379862 0.114525
S -1.502179 0.872628 1.782901
Mo -1.359851 -0.795413 0.017056
S 0.048520 -2.003518 1.570928
S 0.039681 -2.846679 -0.274823
S 0.054921 3.437477 -1.503972
S -0.206453 3.971986 0.443113
S -3.386479 -2.040499 0.633040
S -3.079937 -1.692072 -1.385710
S 3.464691 -1.984489 0.224630
S 2.896234 -1.465106 -1.687672
H 3.392206 -0.033019 1.984196

Isomer O, wB97XD
E= -5381.913185
S 0.858281 -1.395436 -0.718545
Mo 3.008797 -0.550678 0.119527
S 4.623850 0.824643 -1.015524
S 3.312940 -0.241253 -2.200892
S 1.470298 -0.087296 1.888680
S 3.106984 1.155919 1.794572
Mo -0.449842 0.340210 0.050553
S -0.201980 2.630660 0.695121
S 0.907161 2.317005 -1.017846
Mo -3.109485 -0.478276 -0.116447
S -4.975365 0.226454 1.167853
S -5.016297 0.734748 -0.846613
S -3.232335 -2.509812 -0.636081
S -1.755162 -0.333420 1.769982
S -1.834749 0.593512 -1.729721
S 4.048167 -2.246891 0.736250
H 2.133606 2.225902 -0.448403

Isomer A, B3LYP
E= -5382.158739
S -0.000813 0.000336 1.779419
Mo 1.582756 -0.348045 0.032644
S 0.579995 -1.827340 -1.645113
S 0.886290 -2.794592 0.130294
Mo -1.092920 -1.196298 0.031758
S -2.863531 0.629859 0.127196
S -1.871056 0.411382 -1.647303
Mo -0.489954 1.544327 0.031472
S 1.293163 1.414924 -1.645704
S 1.977112 2.164895 0.129449
S -2.430317 -2.659455 1.411814
S -2.692151 -2.947578 -0.637188
S -1.206661 3.805298 -0.636789
S -1.089575 3.433262 1.412112
S 3.898640 -0.857422 -0.637418
S 3.519581 -0.773718 1.411767
H -0.005853 0.003049 3.132677

Isomer T2, B3LYP
E= -5382.201574
S 0.025147 -0.002012 1.817437
Mo -1.546751 -0.003071 -0.004156
S -0.978737 1.693441 -1.656476
S -1.460243 2.510795 0.163558
Mo 0.816299 1.371517 0.013169
S 2.919666 0.004937 0.068192
S 1.920768 0.003953 -1.722035
Mo 0.822356 -1.368809 0.013201
S -0.966082 -1.692437 -1.660018
S -1.453761 -2.517120 0.157649
S 1.789822 3.000821 1.488828
S 2.037385 3.458552 -0.534071
S 2.050718 -3.452552 -0.532067
S 1.800198 -2.993931 1.490446
S -4.022354 -0.020433 -0.643017
S -3.668888 0.083305 1.393807
H -3.758197 -1.221869 1.751280

Isomer T1, B3LYP
E= -5382.205143
S -0.018181 -0.004990 1.808468
Mo 1.535165 -0.237003 0.001789
S 0.695113 -1.801111 -1.684208
S 1.058523 -2.723642 0.118246
Mo -1.012709 -1.238719 0.006389
S -2.880225 0.431287 0.097591
S -1.922844 0.285542 -1.709168
Mo -0.604909 1.470664 0.009973
S 1.190073 1.504916 -1.683103
S 1.828237 2.277064 0.115839
S -2.205651 -2.708422 1.490965
S -2.548275 -3.105484 -0.530596
S -1.520977 3.709233 -0.523194
S -1.305884 3.223473 1.496895
S 4.084436 -0.604057 -0.495879
S 3.485578 -0.513732 1.494660
H 4.424236 0.691209 -0.706591

Isomer B, B3LYP
E= -5382.158636
S -0.108046 0.028767 -1.842675
Mo 1.343798 -0.757260 -0.053766
S 2.267408 0.555926 2.000018
S 2.391425 1.569775 0.162104
Mo -0.113901 1.619036 -0.098360
S -2.601582 1.422986 0.109654
S -1.514116 0.910991 1.797332
Mo -1.393125 -0.795179 0.019626
S 0.031976 -2.014433 1.603217
S 0.007616 -2.890039 -0.248411
S 0.107572 3.482611 -1.511293
S -0.173504 4.023293 0.443548
S -3.447637 -2.026234 0.636786
S -3.123303 -1.679883 -1.398647
S 3.485151 -2.050265 0.194007
S 2.888613 -1.503280 -1.720098
H 3.470413 -0.080512 1.956333

Isomer A, M06
E= -5381.696964
S -0.000653 0.000098 1.789804
Mo 1.564838 -0.368530 0.036271
S 0.549567 -1.824603 -1.636541
S 0.838156 -2.786084 0.129654
Mo -1.101625 -1.170715 0.035527
S -2.832177 0.667167 0.128729
S -1.855062 0.436855 -1.637494
Mo -0.463310 1.539172 0.035473
S 1.306410 1.387899 -1.636516
S 1.993534 2.119286 0.129950
S -2.459851 -2.614190 1.393038
S -2.712634 -2.884420 -0.641348
S -1.140375 3.791589 -0.641254
S -1.035169 3.436918 1.393263
S 3.853734 -0.907043 -0.642031
S 3.494995 -0.823366 1.392685
H -0.003530 0.001381 3.143574

Isomer T2, M06
E= -5381.741559
S 0.025315 -0.001964 1.822606
Mo -1.538601 -0.006140 0.000253
S -0.979896 1.681682 -1.647697
S -1.450800 2.489307 0.163457

Mo 0.806555 1.364497 0.013407
S 2.901555 0.010839 0.069190
S 1.908837 0.007633 -1.708731
Mo 0.817950 -1.358591 0.013529
S -0.960777 -1.685005 -1.651304
S -1.433281 -2.501711 0.157981
S 1.775256 2.994148 1.472773
S 2.014696 3.437189 -0.536299
S 2.041784 -3.423032 -0.534098
S 1.797771 -2.979959 1.474563
S -3.987514 -0.028894 -0.641637
S -3.646926 0.077614 1.380031
H -3.704297 -1.235748 1.724694

Isomer T1, M06
E= -5381.744950
S -0.017125 -0.005137 1.814650
Mo 1.522674 -0.269766 0.005812
S 0.654845 -1.808080 -1.673025
S 0.987993 -2.724397 0.122154
Mo -1.030647 -1.209219 0.007645
S -2.852947 0.489559 0.099173
S -1.904995 0.324678 -1.695409
Mo -0.566928 1.472524 0.010582
S 1.222348 1.470679 -1.672251
S 1.856086 2.218472 0.122327
S -2.254967 -2.651977 1.474344
S -2.594112 -3.027445 -0.533924
S -1.428926 3.711737 -0.527082
S -1.230353 3.239317 1.479748
S 4.017658 -0.675742 -0.507090
S 3.469753 -0.583871 1.478532
H 4.341721 0.626678 -0.723991

Isomer B, M06
E= -5381.693750
S -0.099624 0.028849 -1.848734
Mo 1.303686 -0.807251 -0.047416
S 2.212883 0.498612 1.965989
S 2.462739 1.436001 0.123553
Mo -0.043074 1.613956 -0.102558
S -2.521924 1.517642 0.096005
S -1.471719 0.967620 1.777242
Mo -1.414049 -0.733594 0.013916
S -0.055202 -2.003516 1.598056
S -0.111273 -2.866873 -0.243807
S 0.250867 3.484968 -1.485959
S 0.005911 3.980859 0.468240
S -3.502139 -1.872091 0.628182
S -3.170929 -1.548862 -1.391904
S 3.366342 -2.167448 0.232217
S 2.825251 -1.635004 -1.686255
H 3.385488 -0.202731 1.989200

References

[1] N. K. Bersenkowitsch, M. Ončák, J. Heller, C. van der Linde, M. K. Beyer, *Chem. Eur. J.* **2018**, *24*, 12433.
[2] A. Herburger, C. van der Linde, M. K. Beyer, *Phys. Chem. Chem. Phys.* **2017**, *19*, 10786.
[3] A. Müller, V. Wittneben, E. Krickemeyer, H. Bögge, M. Lemke, *Z. Anorg. Allg. Chem.* **1991**, *605*, 175.
[4] A. Baloglou, M. Ončák, M.-L. Grutza, C. van der Linde, P. Kurz, M. K. Beyer, *J. Phys. Chem. C* **2019**, 8177.
[5] a) M. J. Frisch, G. W. Trucks, H. B. Schlegel, G. E. Scuseria, M. A. Robb, J. R. Cheeseman, G. Scalmani, V. Barone, G. A. Petersson, H. Nakatsuji et al., *Gaussian 16 Revision B.01*, **2016**; b) M. J. Frisch, G. W. Trucks, H. B. Schlegel, G. E. Scuseria, M. A. Robb, J. R. Cheeseman, G. Scalmani, V. Barone, G. A. Petersson, H. Nakatsuji et al., *Gaussian 16, Revision A.03*, Gaussian Inc., Wallingford CT, **2016**.
[6] Computational Chemistry Comparison and Benchmark Database. NIST Standard Reference Database Number 101, http://cccbdb.nist.gov/, last accessed 5.10.2020.
[7] T. Shimanouchi, Tables of molecular vibrational frequencies. Consolidated volume I, National Bureau of Standards, Washington, D.C., **1972**.
[8] E. Isoniemi, L. Khriachtchev, M. Pettersson, M. Räsänen, Chem. Phys. Lett. 1999, 311, 47.
[9] I. W. May, E. L. Pace, Spectrochim. Acta A **1968**, 24, 1605.

Author Contributions

AB: Experimental planning and investigation (lead), data analysis (lead), results discussion (equal), preparation of the manuscript (lead).
MP: Computations (supporting), results discussion (equal).
MO: Computations (lead), results discussion (equal), manuscript editing (lead).
MLG: Chemical synthesis (lead), results discussion (equal).
PK: Project supervision and funding acquisition (lead), results discussion (equal), manuscript editing (supporting).
MKB: Conception of original idea, project supervision and funding acquisition (lead), data validation (lead), results discussion (equal), manuscript editing (lead).
